# Knowledge and practices of parents about child eye health care in the public sector in Swaziland

**DOI:** 10.4102/phcfm.v10i1.1808

**Published:** 2018-11-07

**Authors:** Velibanti N. Sukati, Vannesa R. Moodley, Khathutshelo P. Mashige

**Affiliations:** 1 Discipline of Optometry, University of KwaZulu-Natal, South Africa

## Abstract

**Background:**

Swaziland, like many other developing countries, lacks appropriate eye health services, particularly for children.

**Aim:**

To determine the knowledge and practices of parents about child eye health care in the public sector in Swaziland.

**Setting:**

The setting for this study was Swaziland.

**Methods:**

A descriptive study involving cross-sectional sampling methodology and quantitative analysis was employed with 173 randomly selected parents whose children attended public schools in Swaziland.

**Results:**

Out of 173 participants, 104 (60.1%) parents reported that they have never taken their children for an eye test and 69 (31.7%) felt that their children’s vision was fine. Ninety-seven (53.1%) parents indicated having no knowledge about child eye conditions and no significant association was found between level of education and knowledge of eye conditions affecting children (*p* = 0.112). Having an immediate family member who wore spectacles increased the likelihood of a child being taken for eye testing (*p* = 0.001), but decreased the likelihood of being well informed about eye health (*p* = 0.218). Of those parents who reported taking their children for eye tests, 34 (49.3%) reported that they were given eye drops and 31 (44.9%) stated that their children were prescribed spectacles. Eighty-seven (50.3%) parents accepted the idea of their children wearing spectacles.

**Conclusion:**

The findings of the study suggest the need for parents to be informed about basic child eye health care and the importance of their children having regular eye examinations.

## Introduction

Visual impairment and blindness in children from the developing world are usually caused by avoidable and treatable conditions.^1,2^ These limit their access to education and job opportunities impacting negatively on their productivity and quality of life.^1,2,3^ A major contributing factor to childhood blindness is a lack of awareness about promotive and preventative eye care measures among parents or guardians and community members as well as knowledge of where to access appropriate care.^2,4^ Approximately 500 000 children become blind each year, with 1.5 million already blind, five times higher in the poor regions compared to affluent regions. The situation is so severe that it is estimated of every minute a child goes blind, with 60% dying within a year after becoming blind.^5^ An estimated 1.3 million blind children live in Africa (18%),^6,7^ with refractive error (RE) being relatively low (1.8%) in most African countries, reportedly too low to justify the prioritisation of RE screening only.^8^

Studies about awareness of eye conditions have been conducted in developed and developing countries and concluded that many seek timely eye care to reduce the burden of blindness, even among children.^9^ Muhit et al.^10^ and Shrestha et al.^11^ reported that poor health-seeking behaviour among parents emanated from poor health literacy. A recent study from Sudan by Alrasheed et al.^12^ also concluded that parents were against the use of spectacles by their children. The authors concluded that, as a public health goal, advocacy for parents’ education was necessary. Other reasons highlighted for the non-utilisation of services were related to socio-economic or personal factors.^1,2,4,13^ The lack of human resources and facilities and ineffective policies have also been suggested as possible causative factors, particularly in low-income countries.^2^

The absence of programmes aimed at identifying children who require examination, treatment, referral or rehabilitation in communities and schools adds to the cost burden on parents, guardians and caregivers.^14^ Although the number of children in Swaziland form a significant part of the overall population, challenges still remain regarding access to eye health because of the absence of relevant health and eye care services as well as policies. It is, therefore, anticipated that there will be knowledge inadequacy among parents about child eye health which will impact on the lack of understanding of the importance of early detection and intervention. This knowledge gap is underpinned by the inadequate promotive and preventative interventions associated with weak health systems resulting in poor performing health care services in the country suggesting the urgent need to improve eye health literacy among the population. Furthermore, the inclusion of parents, guardians, legal caregivers and local chiefs when developing ophthalmic services for children and having child eye health education focused on them is essential. It would, therefore, be necessary to evaluate the knowledge and practices of parents regarding child eye health care in order to assist policy-makers to create and implement effective eye health plans.

The health system of Swaziland consists of both formal (regulated private and public health services) and informal (unregulated) sectors. Most of Swaziland’s 1.1 million citizens rely on the public sector for health care services, including eye health care with an estimated blindness prevalence of approximately 1%.^15,16^ Out of the total population, children account for almost half (44%), making a focus on their health, specifically eye care, essential, so that they can access education and become productive adults.^15,16^ The national statistics revealed that in 2008, 16.8% of the population were disabled, of whom 63.1% had visual impairment, followed by 15% with hearing difficulties.^16^ Swaziland suffers from a brain drain of eye health work force with 1 ophthalmologist, 1 cataract surgeon, 5 optometrists and 13 ophthalmic nurses unevenly distributed in the 4 districts of the country. There are few public sector eye clinics, and spectacles are provided throughout the country. Cases requiring more specialised attention are referred to South Africa, which requires the funding from government for those who cannot afford to be referred and attended to.

## Methods

The study employed a descriptive study involving cross-sectional sampling methodology and quantitative analysis using a validated questionnaire to investigate the knowledge and practices of parents about child eye health care in the public sector in Swaziland. Prior to preparing the sampling frame, the support of local education authorities and school officials was sought. The questionnaire was pilot tested on respondents that were not included in the final study sample, and a statistician provided support with the use of appropriate data analysis techniques. The study population consisted of parents whose children attended public schools in two of Swaziland’s four regions purposively selected for the study: Hhohho, a more affluent urban region, and Lubombo, a rural and poor region of Swaziland. The sampling frame consisted of all 110 and 200 public schools in the Hhohho and Lubombo districts, respectively.

The semi-structured questionnaire consisted of three sections: general and demographics questions, knowledge about eye care and practice about eye care. The questionnaires were distributed by trained research assistants who were knowledgeable with the content of the questionnaires. Written informed consent forms were obtained from each participant after familiarising themselves with what the study entailed. All questions requiring positive and negative responses were followed by a space to allow parents to state reasons why a particular response was chosen to eliminate errors and bias. Ten children were selected randomly in 35 primary and 7 secondary schools to get a total of 170 parents. The inclusion criteria consisted of parents of selected children in public schools and excluded parents of children of non-selected children in public schools. Using a 95% probability and assuming 50% of parents were knowledgeable, the minimum sample size was calculated to be 170. The quantitative data were captured and analysed using the Statistical Package for Social Sciences (SPSS version 24) in consultation with a statistician. Findings are presented as descriptive statistics, tables and graphs. The statistical analysis included frequency counts, cross tabs and chi-square tests for correlations. Statistical significance was set at a 95% confidence interval, and all *p*-values less than 0.05 were considered statistically significant. The data will be stored in a locked cupboard for 5 years after which it will be shredded.

## Ethical considerations

Participation in the study was voluntary as stipulated in the University of KwaZulu-Natal (UKZN) consent form, and only those who completed consent forms were allowed to participate in the study. All data captured were kept confidential, and no subject was identified by name. The study proposal was approved by the Biomedical Research and Ethics Committee of University of KwaZulu-Natal (BE338/13), the Swaziland Health Ethics Committee (MH/599C) and the Ministry of Education.

## Results

### Demographic characteristics of parents

A total of 173 questionnaires were completed by parents of children attending government schools in the Hhohho and Lubombo regions. Of those who completed the questionnaires, 117 (67.6%) were females and 56 (32.4%) were males. Ninety-three (53.8%) parents reported having acquired a tertiary qualification, 65 (37.6%) had obtained between Grades 6 and 12, 11 (6.4%) went up to Grade 6 and 4 (2.3%) reported that they have never attended school. Fifty-four parents (31.2%) were civil servants, and the occupations of other parents are shown in Figure 1.

**FIGURE 1:**
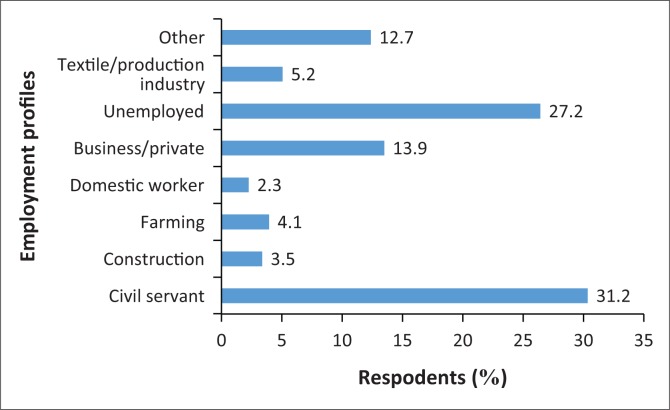
Parents’ employment profiles.

One hundred and fourteen parents (65.9%) indicated that they had no immediate family members who wore spectacles and 59 (34.1%) did. Sixty parents (34.9%) indicated that they took more than an hour to get to the nearest health facility, 52 (30.1%) took 31‒60 min, 41 (23.7%) took 16‒30 min and 20 (11.6%) took 0–15 min. The time taken to the nearest facility was significantly associated with the occupation of parents (*p* = 0.037) but insignificantly associated with their level of education (*p* = 0.403). Furthermore, the time taken influenced the type of facility utilised by parents for their children’s eye examination (*p* = 0.018). Sixty-three (36.4%) parents reported using the bus, 50 (28.9%) used mini buses or taxis, 37 (21.4%) used private transport and 23 (13.3%) walked to the health facility. Respondents who indicated using buses, mini buses and taxis as well those who walked were more likely to utilise public eye care facilities (*p* = 0.004). The most common type of social media used by parents was WhatsApp (50.3%). Other responses regarding the use of social media are shown in Appendix 1.

**FIGURE 2:**
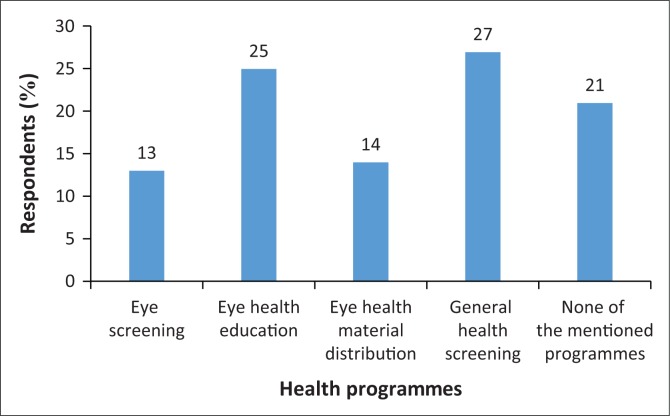
Parents who reported health programmes in their home area.

Fifty-four parents (27%) reported that general health screenings were conducted in their communities, and 26 (13%) reported that eye health materials were distributed in their areas. Other reported health programmes are shown in Figure 2.

### Parents’ knowledge about eye care

One hundred and four (60.1%) parents reported that they have never taken their children for an eye test, while other responses regarding child eye care are illustrated in Table 1.

**TABLE 1:** Parents responses about their knowledge of eye care.

Questions asked	Yes		No	
	*n*	%	*n*	%
Since your child and/or children was or were born, have you ever taken him or her for an eye test?	69	39.9	104	60.1
If yes, was it before school?	24	34.8	45	65.2
If not, what were the reasons?	-	-	-	-
Did not know my child has to be tested	-	-	30	28.9
Did not see the need	-	-	19	18.3
Child was seeing well	-	-	33	31.7
Financial constraints	-	-	8	7.7
No eye care facilities available	-	-	6	5.8
Only old people experience eye problems	-	-	8	7.7

The level of education was not significantly associated with having an eye test (*p* = 0.472), before schooling (*p* = 0.302) or the reasons for not taking child for eye testing (*p* = 0.067). Forty (58%) parents reported that they took their children for the first time for vision testing when they were 10 years and older, 12 (17.4%) reported that they took them when they were 6 years old, 12 (17.4%) reported that they took them when they were 6 months old and 5 (7.3%) reported taking their children when they were 3 years old. The age at which the child was taken for an eye test was insignificantly associated with the level of education of the parents (*p* = 0.161); however, having an immediate family member who wore spectacles influenced early testing (*p* = 0.013). Rubbing eyes repeatedly and suspicion of visual problems were the main factors that prompted parents to take their children for eye testing. Other factors that prompted parents to take their children for eye testing are shown in Appendix 2.

Thirteen parents (7.5%) reported having children who had to drop out of school because of poor vision. Having an immediate family member who wore spectacles increased the likelihood of a child being taken for eye testing (*p* = 0.001), but was not significantly associated with being well informed about eye health (*p* = 0.218). One hundred and twenty-seven (73.4%) parents agreed that poor vision could affect their children’s performance at school and 23 (13.3%) each disagreed or were unsure. One hundred and eleven (45.3%) parents reported that eye diseases were the cause of poor vision, and 62 (25.3%) parents felt that watching television (TV) was the most common cause of poor vision (Appendix 3).

Ninety-seven (53.1%) parents indicated having no knowledge about childhood eye conditions, and no significant association was found between the level of education and knowledge of eye conditions affecting children (*p* = 0.112). Of those who indicated being knowledgeable about childhood eye conditions, 44 (32.8%) reported that they knew about refractive errors and 37 (27.6%) reported that they knew of allergic conjunctivitis. Other responses regarding knowledge of eye conditions affecting children are illustrated in Appendix 4.

Forty-two (31.3%) parents indicated that medical doctors provided them with information about eye diseases. Other responses regarding the source of information about eye care knowledge are shown in Appendix 5.

Appendix 6 shows that 102 (59%) parents reported that they have never had any eye health professionals visit their area or community. Of those who reported that eye health professionals had visited their area, 19 (47.5%) and 10 (25%) indicated that the last visit by the eye health professionals was less than a year and between 2 and 4 years, respectively. Nineteen (43.2%) and 10 (22.5%) parents, respectively, reported that optometrists and allied health workers were part of these visits (Appendix 6).

**FIGURE 3:**
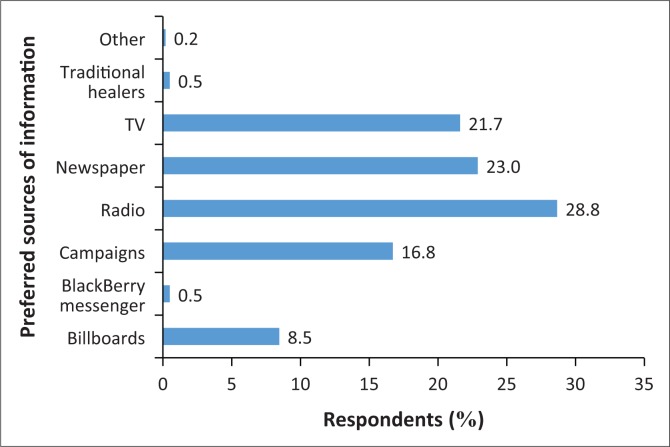
Response of parents regarding preferred sources of information.

One hundred and thirteen (65.3%) parents reported that they lived in an area that had a health clinic, 52 (30.1%) did not and 8 (4.6%) were unsure if there was a health clinic in the area they lived in. The knowledge of existing public facility was significantly associated with the level of education (*p* = 0.006) of the respondents and where a child was taken to receive eye care (*p* = 0.005). In addition, the last visit by eye health professionals was also found to be insignificantly associated with having a clinic facility in the area (*p* = 0.100). Of those who reported that they had a clinic in their area, 56 (47.5%) indicated that the clinic did not offer eye care services, 42 (35.6%) reported that eye care services were provided and 20 (16.9%) were unsure. The presence of a health facility in a community had an influence on early child eye examination (*p* = 0.001). One hundred and three (59.5%) parents reported that they were not well informed about eye health, 40 (23.1%) felt that they were and 30 (17.3%) were unsure. Being knowledgeable about eye health was insignificantly associated with the level of education of respondents (*p* = 0.086) and early child eye examination (*p* = 0.061) and eye health professionals’ visits (*p* = 0.070). Figure 3 shows that the majority of parents felt that mainstream media, which included radio, newspaper and TV, was their preferred option to spread information about children’s visual problems.

Eighty-seven (50.3%) parents accepted the idea of their children wearing spectacles, 43 (24.8%) were either unsure or did not agree that children should wear spectacles. Educated parents reported that their children wore spectacles and were informed about the purpose of wearing them; however, no significant association was found between having spectacles and the purpose of wearing them (*p* = 0.834 and *p* = 0.651, respectively). Seventy-seven parents (44.5%) indicated that wearing glasses at an early age was not a consequence of poor vision, 60 (34.7%) were unsure and 36 (20.8%) agreed. In terms of equal access to educational opportunities for blind children, 58 (33.5%) parents strongly agreed with this notion, 52 (30.1%) agreed, 43 (24.9%) were unsure, 14 (8.1%) disagreed and 6 (3.5%) strongly disagreed. Other responses regarding eye care knowledge and education of visually impaired or blind children are shown in Table 2.

**TABLE 2:** Response of parents regarding eye care knowledge and education of visually impaired or blind children.

Questions and/or items	Yes		No		Unsure	
	*n*	%	*n*	%	*n*	%
In your immediate family are there any children who were born blind or visually impaired?	20	11.6	133	76.9	20	11.6
If yes, was the cause of blindness or visual impairment explained to you by the doctors?	19	95.0	1	5.0	-	-
Does the visually impaired and/or blind child and/or children attend school?	13	65.0	7	35.0	-	-
If not, why?	-	-	-	-	-	-
Do not know where to send child and/or children for schooling	-	-	1	20.0	-	-
Without seeing you cannot be educated	-	-	2	40.0	-	-
Scared that the child and/or children will not cope	-	-	2	40.0	-	-
Child and/or children will be teased by peers	-	-	-		-	-
Other	-	-	-		-	-
Do you know any school for the blind and/or visually impaired in the country?	121	69.9	52	30.1	-	-

One hundred and thirty-three (76.9%) parents indicated that none of their children were born blind or visually impaired, 20 (11.6%) each were unsure or agreed that their children were born blind or had a visual impairment. Of those who agreed, 19 (95%) reported that medical doctors explained the cause of blindness. Of the parents who indicated that their children were born or had gone blind, 16 (80%) of those had no knowledge of the onset of visual loss, 2 (10%) indicated that it was within the first year of life and another 2 (10%) reported it to have occurred when the children were aged 2–15 years.

### Parents’ practices about eye care

Thirty-four parents (49.3%) reported that their children were dispensed eye drops when they visited the eye clinic and 31 (44.9%) reported that they were given spectacles. Two (2.9%) parents reported that no action was taken when they visited a health facility and 2 (2.9%) reported that they were referred for further care. Twenty-four (34.9%) parents took their children to private optometrists, 23 (33.3%) to public hospitals and 22 (31.9%) to public clinics. Parents who took their children to private optometrists indicated being well informed about eye health compared to those who took their children to public facilities, and this association was found to be statistically significant (*p* = 0.049). One hundred and thirty-two (76.3%) parents reported that their children did not wear glasses. There was no significant difference found between parents who agreed to their children wearing spectacles (*p* = 0.118) or undergo surgery (*p* = 0.454) and those who did not, compared to those who indicated being well informed about eye health. Of those who reported that their children wore glasses, 30 (73.2%) knew what they were for. Table 3 shows the other responses regarding practices about eye care.

**TABLE 3:** Responses by parents regarding practices about eye care.

Questions and/or items	Yes		No		Unsure	
	*n*	%	*n*	%	*n*	%
Do any of your children wear glasses?	41	23.7	132	76.3	-	-
If yes, do you know what they are for?	30	73.2	-	-	11	26.8
Will you have a problem if your child and/or children had to wear glasses?	31	17.9	131	75.7	11	6.4
Would you allow your child and/or children to undergo eye surgery?	81	46.8	57	33.0	35	20.2
If not, why?	-	-	-	-	-	-
Fear of the outcome	-	-	19	31.7	-	-
Damage eye more	-	-	20	31.3	-	-
Cost of the actual operation	−12	-	18.8	18.8	-	-
Knowledge of services	-	-	10	15.6	-	-
Cultural and social barriers	-	-	2	3.1	-	-
Accessibility to services	-	-	1	1.6	-	-

## Discussion

### Demographic profiles

Many parents reported taking a long time to reach the nearest eye health facility, which could be because of the fact that they are few eye clinics in Swaziland, as well as factors such as the socio-economic climate, a scarcity of human resources and poor road infrastructure. Ebeigbe and Emedike^17^ reported that the provision of eye care was dependant on parents’ knowledge of children’s ocular conditions as this impact on their eye care-seeking behaviour because of the dysfunctional structure of the levels of care to detect and manage childhood blindness in Nigeria. The educated parents who participated were often civil servants who generally resided in urban areas where eye care facilities are available, public transport is accessible or they have their own private transport. However, many more of the parents were unemployed, which is in line with the 2005 Ministry of Economic Planning and Development report.^18^ The literacy rate among parents was also in line with 2006 census report at 78%.^15^ A high utilisation of public eye care facilities was noted among parents who rely on public transport, which may be related to their varying socio-economic status. Regardless of the time taken, most parents took their children to public facilities for eye testing.

The results on social and mainstream media suggest that the spread of information about eye health can be enhanced, as many respondents indicated having access to social media. However, increased access was noted among parents who had a tertiary qualification compared to those who did not. Parents who had eye health education in their communities reported being well informed compared to those who indicated having access to vision screening and eye material distribution. Mbonye^19^ reported high perception of parents about childhood diseases; however, practices towards eye care remained poor in rural Uganda.

This highlights the importance of eye health education, even when vision screening services and eye health material distribution are offered at community level. The lack of eye health-related activities, such as vision screening in communities, suggests the need to prioritise them. The lack of vision screening could also be because of the fact that other health-related programmes, such as nursing and medicine, which are perceived as ‘more essential’ services, are prioritised. Therefore, integrating eye health services into the primary health care (PHC) programmes will circumvent the isolation of ophthalmic services.

### Parents’ knowledge about eye care

Many parents had never taken their children for an eye test for various reasons, such as the belief that their children’s vision was adequate, a lack of knowledge about regular eye tests and not seeing the need to do so. This could be because of poor knowledge about health care in general and eye health care in particular, which results in low utilisation of eye care services. Shrestha et al.^20^ reported that poor health-seeking behaviour among parents for their children was a result of poor health literacy. Parents with less education reported that they did not see the need for their children to have an eye test, with only a few with tertiary qualification reporting such results. This suggests that the level of education may play a significant role in the knowledge of their children’s eye health. Furthermore, respondents with less education experienced more financial constraints and a greater lack of available eye care services than those who had tertiary qualifications. This could be because of those with tertiary education being able to afford to take their children for eye testing as they are employed and possibly reside in areas where eye care facilities are available. Again, the less educated parents demonstrated less knowledge regarding reasons for not taking their children which is supported by the fact that most indicated that only old people experience eye problems. Eye health education targeting all parents may help to improve eye care-seeking behaviour for their children. Taking children for a comprehensive examination before admission to school needs to be a requirement at primary school level in order to redress visual deficits cases. In doing so, many children will have had an eye test in the first 6 years of their life, and appropriate early recommendations will be made for them rather than waiting for years to realise a child fails to cope because of visual problems.

A cross tabulation between education level and eye test revealed that highly educated parents were more likely to take their children for an eye test than those who were less educated. However, few parents took their children for eye testing before they started schooling. While parents with higher levels of education would be expected to know the importance of early intervention to avoid visual problems that can lead to amblyopia and visual impairment or blindness, the results show that the level of education was not a determinant for parents taking their children for eye testing. This points to the need for all parents, regardless of education level, to be informed about the importance of early intervention among children to narrow the gap between those at an advantage to receive eye services and those who do not, regardless of their socio-economic status in society.

This study found that parents waited for warning sign or symptoms before taking their children for eye health care, which could suggest that they were not knowledgeable about preventative eye care issues that affected children. Nirmalayan et al.^20^ found that respondents did not see the need of having periodic eye examinations unless the child complained, or parents identified the problem. Frazer and Kleinstein^4^ and Owsley et al.^21^ identified the lack of knowledge as a factor that influences parents from taking sound decisions about preventing visual disorders as well as systemic conditions that have visual consequences. However, some parents (albeit few) in this study indicated that they took their children to an eye care facility after suspecting vision problems or were referred by general practitioners. This indicates that some parents did take action when they suspected vision problems. However, there is a need to promote preventative programmes for children, which can be conducted as part of general wellness programmes that target parents or guardians.

The low referral rate by medical doctors may be because of the non-utilisation of health care facilities and lack of eye care services to which to make informed timely referrals. However, our study found that people rely on doctors and nurses for eye health information. The fact that teachers did not receive adequate eye health training from their training college, coupled with the ineffective school health programme, resulted in them being unable to identify children with visual problems and give proper advice to parents. Parents’ range of education levels took their children for eye testing at a late stage in life, 6–10 years and over, regardless of having an immediate family member wearing spectacles. However, having an immediate family member who wore spectacles influenced the eye-care-seeking behaviour among parents regardless of their education level. This could possibly be because of previous exposure to eye health services and parents being cautious. Respondents who had access to social and mainstream media took their children for eye testing, suggesting that these media instruments play an important role in spreading eye health information about available eye services. Rubbing of eyes repeatedly prompted parents across all levels of education to take their children for eye testing, which is a positive result. However, there are other eye conditions that do not have obvious signs that need to be diagnosed at an early age before they cause irreversible damage, highlighting the importance of child eye health education focused on parents or guardians, legal caregivers and local chiefs, as well as formulating proper communication linkages.

Parents with higher education levels demonstrated better knowledge of eye conditions that affect children than those with lower education levels. However, conditions such as congenital cataracts were unfamiliar to all the parents. This suggests that the level of education might not necessarily indicate that parents are well informed about the important eye health issues affecting children. A few parents indicated that their children dropped out of school because of visual problems. Similarly, several parents indicated having children in their immediate family who were blind. These few cases require intervention as they are usually marginalised and denied their basic rights to access health, education and social services, as asserted by Corn et al.^22^ and Jones.^23^ There is therefore a need to ensure that these children receive all the support from their parents or guardians and communities, as available resources, such as the school for the blind, are available to cater to their specific needs. Many parents reported that eye care practitioners explained the causes of visual impairment or blindness to them.

Barriers that may arise from this exercise, such as language, attitudes and culture, need to be addressed by eye health professionals. This will assist parents to probably make informed decisions about the situation affecting their children. Many parents failed to note the age of onset of vision loss for their children, with early identification of visual problems often leading to a reduction in avoidable blindness. Frazer and Kleinstein^4^ reported that even among the most perceptive parents, children who have a visual problem can often go unnoticed until a late stage when damage cannot be reversed. Factors such as poor eye health literacy, socio-economic status or prioritising other health-related illnesses among parents, guardians and the community at large are some of the reasons for the non-utilisation of readily available ophthalmic services for their children. Therefore, early intervention can be achieved by investing in awareness campaigns on promotive and preventative eye care to improve eye health-seeking behaviour among parents or guardians.

Most educated parents knew that blind children need to attend the school for the blind. Some educated parents highlighted that they did not know where to send their children for schooling and were scared that their children would not cope, while those with less education indicated that blind children cannot be educated. These reports from parents showed that negative perceptions about blind children and education exist among parents regardless of their education level. A school report from South Africa^24^ highlighted the fact that parents played a role in preventing blind children from attending school because of certain perceptions or beliefs. These include the perception that without good vision the child cannot be educated, blind children cannot cope at school, parents lacked knowledge where to send the child and existence of special schools, fear of children safety and the high cost of enrolment.^24^ In cases where parents were informed about existing facilities for the blind, limited space was a challenge for many children. It is suggested that the government up-scale interventions to assist children living with visual disabilities by increasing the number and capacity of blind schools to take in more children as well as equipping teachers with relevant skills to teach the visual impaired and blind.

Although many parents reported that poor vision affected their children’s school performance, a significant proportion thought that it did not. These findings suggest the need for awareness campaigns among parents regarding the possible negative effects of poor vision on school performance. Some parents knew that eye diseases can cause poor vision, as well as factors associated with watching TV, and might have observed their children moving closer to the TV or peering. Computer games, headaches and reading were also reported as causes of visual problems by parents. These may indicate the presence of RE or other ocular problems that manifest when performing visually demanding tasks. Therefore, the spread of information about available eye care services needs to be emphasised to parents, together with public health messages about preventive eye care that is aimed at increasing eye heath utilisation and literacy.^25,26,27^

The lack of knowledge about childhood eye diseases may hamper early interventions that could potentially prevent vision loss. Reports by Frazer and Kleinstein,^4^ Owsley et al.,^21^ Flores and Vega^28^ and Robin et al.^29^ suggested that early intervention is important in providing good visual outcomes. The authors concluded that the lack of health literacy could explain how health care information can be disregarded as well as prioritising other health services without understanding the importance of a child to have a vision examination is a cause for concern. This study also reports similar findings of knowledge inadequacy about eye health among parents.

Eye health education appears to be a neglected subject in health promotion efforts, possibly because of the weak eye health system in Swaziland. This requires all relevant stakeholders, including the government and non-governmental organisations (NGOs), to address poor eye health literacy. Most parents reported that they knew about eye conditions such as RE, allergic conjunctivitis and vitamin A deficiency (VAD). Exposure to eye health care and the prioritisation of RE services^10,11^ are possible reasons for these results. In addition, most parents had some level of education, which plays a vital role in influencing how information can be obtained, understood and shared.^4,28^ A few parents indicated that congenital cataracts were among the major causes of blindness among children, which could be because of the fact that this type of cataract is removed early in children. Gogate et al.^30^ argue that established links among health care providers and within communities increase the numbers of children in need of cataract surgery at an early age because of improved problem identification. Parents need to be perceptive about their children visual status and ensure that they go for periodic eye examinations. Advocacy for parents through eye health education in communities and media campaigns as a public health goal is therefore necessary.

The fact that few health professionals were reported as the main source of information about eye conditions may be because of the lack of human resources in eye health.^4,29^ Parents reported mainstream media, such TV, radio and newspaper, to be their main sources of information about eye health. These mainstream media are accessible to most parents and should be encouraged to continue delivering positive messages about child eye health and inform communities about where eye care services can be accessed. Many authors^31,32,33,34^ have indicated the use of mainstream media as a powerful tool to spread eye health information targeting mostly those in impoverished areas that lack eye care facilities. However, only those who could afford such items can utilise this privilege making it important for government to offer affordable eye health in the country.

Many parents reported that they have never had a health official visiting their communities, which could be because of the fact that the majority of the population live in rural areas, where eye health and human resource services are scarce. Ali et al.,^35^ Kawuma and Mayeku^36^ and Kello and Gilbert^37^ reported similar results. Those parents who reported that eye health professionals visited their areas may be located in urban areas where there are eye health facilities. Many parents indicated that optometrists were the highest number of health professionals during these visits to conduct outreach. As there are currently only five optometrists in Swaziland, parents may be confusing other health workers, such as ophthalmic nurses, with optometrists.

Balabanova et al.^38^ suggested that the expansion of the health system was important to improve services; however, it often fails to achieve the desired outcomes. In this study, many educated parents reported having a health facility in their home area, with very few offering eye care services. This lack of eye health services both in urban and rural areas corresponded with the responses of parents who indicated that they were not informed about eye health care, even among those who were educated. Many of those who indicated being well informed about eye health had never taken their children for eye testing before their first year of schooling. These responses could possibly be because of non-exposure and poor eye health-seeking behaviour of parents. It is therefore recommended that eye health services be made available to areas where they are scarce or unavailable by decentralising eye services, coupled with promoting PHC service utilisation, with an emphasis on referral protocols. Training of community health workers in community centres or clinics, facilitated by the eye health professionals, will benefit communities through eye health education, which needs to be continuous to empower health workers to distribute eye health information.

The presence of a health facility in the community was not a determining factor of having eye health professionals visiting the clinic, even though parents reported less than a year visits. These circumstances widen the inequality gap regarding accessing eye health services by children disadvantaging those living in communities without clinics. Priority areas lacking health clinics need to be identified in order to eliminate barriers such as accessibility, geography and affordability.

The closer the health facility is to those who need it, the more likely it would be utilised,^39^ this being confirmed by the study parents residing in communities with health facility who were more likely to take their children for early eye examination. There is a need for public health messages about preventive eye care and diseases aimed at increasing utilisation of eye care services even in communities without health facilities. Parents who reported that eye health professionals visited their areas were not knowledgeable about eye health. This is possibly because promotive and preventive measures in eye health are lacking in the country. In addition, because of the chronic shortage of eye health professionals, clinics do not prioritise the spread of eye health information beyond the clinic walls, highlighting the urgent need of eye health guidelines.

Many authors have advocated the use of electronic devices for accessing health information to address the traditional barriers to access, such as geography, finances and cultural factors.^31,34^ Most parents reported the use of mainstream media to promote public eye health campaigns. An Australian report by Muller et al.^32^ indicated that diabetic patients were informed about ocular consequences of the systemic disease through the use of radio, regional TV and newspaper. Information can also be displayed on billboards to inform communities about priority eye conditions and available eye facilities. A few parents still believed that traditional healers can play a role in spreading information about eye health. This cadre of health workers could be trained by eye health professionals and used as a resource to disseminate information about eye health, as they seem to be more acceptable and trusted in certain communities.

More than half of the parents agreed that spectacle wear by children is good, while 24.8% were unsure and 24.8% reported to the contrary. Nirmalayan et al.^20^ found that respondents in their study believed that all children below the age of 4 years should not be allowed to wear spectacles at all. In our study, more parents with less education indicated having problems with children wearing spectacles, whereas children of educated parents were reported to be wearing spectacles, suggesting that they were more likely to be taken for eye testing. Certain eye conditions may require spectacle wear regardless of the child’s age. Approximately 20.8% of the parents reported that spectacles can worsen vision, with no significant difference in responses between parents of different education levels. This indicates the need for eye health practitioners to explain to parents the benefits and advantages of wearing spectacles.

Overall, one-third of parents both strongly agreed (33.5%) and agreed (30.1%) that children born blind have equal opportunities as sighted children. This suggests that parents do not view blind children as different from other ‘normally sighted’ children. The government needs to be encouraged to make available the infrastructure and human resource support to assist blind children for them to realise their full potential.

### Parents’ practices about eye care

Eye drops were dispensed to most children to treat infections compared to spectacles to correct RE. The low use of spectacles in children reported by parents may be because of the low prevalence of refractive errors, as reported by Wedner et al.^8^ Some parents preferred taking their children to private optometrists to get their eyes tested, while others took them to public hospitals or clinics to access these services. Many parents who indicated being well informed about eye health took their children to a private optometrist rather than public facilities. This may be because of their lack of awareness about the available ophthalmic facilities, as well as the socio-economic status and being able to afford private services. In addition, the lack of eye health professionals and equipment might have influenced parents to take their children to private optometrists. Therefore, eye health awareness programmes are essential to ensure that communities are informed about eye clinics in their area when they are available. While many parents reported being well informed about the purpose of wearing spectacles and did not have a problem with their children wearing them, a few felt that it was not right for children to wear spectacles. This highlights the need to educate parents about the value of spectacle wear for children with visual deficits. Applying eye health programmes as specific interventions for parents by taking advantage of successful health programmes such as ‘national immunisation days’ need to be considered.

A few parents agreed that children may undergo eye surgery when indicated, this being similar to Frazier and Kleinstein.,^4^ Newland et al.,^40^ Shirima and Geneau^41^, who reported that some parents were reluctant to take their children for eye surgery, preferring to wait until they were older. However, delays can worsen the prognosis of an eye condition, many of which, such as congenital cataract, require prompt interventions. This result indicates the lack of knowledge among parents about the need for early interventions to prevent vision loss. These beliefs could be because of varying reasons, including that surgery is prone to damaging the eye, the fear of outcome, cost, as well as poor knowledge about ophthalmic services, among others. A few parents reported culture, social barriers and accessibility to services for not undergoing surgery. This suggests that the availability of a health facility does not automatically translate to access for communities. People need to be informed about eye health and become knowledgeable about why they should make use of such facilities through mainstream and social media awareness campaigns. Parents need to monitor children’s visual status from an early age, as early treatment is more likely to lead to good prognosis than later in life.

The study findings also highlight that being well-informed about eye health does not guarantee best practices by parents, as they were less likely to allow their children to wear glasses and undergo surgery, while other studies reported the contrary.^4,10,11,42^ Poor utilisation and exposure because of a lack of ophthalmic services and human resources are possible reasons for the findings in this study. There is a need to educate the public about the importance of regular eye examination and the consequences of delayed eye health care.

The limitations of this study included the following:

- The study was conducted in two of the country’s four regions, and because of the diversity in characteristics of different communities and other social determinants of health, the findings may not be generalised to the whole country.
- The sample of parents may not be a representative of all parents’ views regarding children’s eye health knowledge and practices.

Based on the limitations and findings of this study the following recommendations are made:

- Conduct large surveys, preferably on a national basis, to provide sufficient data to increase public awareness and expedite appropriate measures to curb visual impairment and blindness among children.
- Investigate knowledge differences between urban and rural parents on barriers to eye health care among children.
- Investigate the impact of socio-economic classes of parents’ knowledge about eye health compliance for their children.

## Conclusion

The findings of the study suggest the need for parents to be informed about basic child eye health in order to seek appropriate care promptly. Eye health education is a neglected subject as both educated and uneducated parents showed poor knowledge about child eye care. These suggest the need for all parents in communities to be well informed about child eye health. As many people rely on public eye health facilities, the government must ensure that quality ophthalmic services are rendered in all public eye clinics to curb any preferences for facilities leading to disregarding referral protocols. It is, therefore, recommended that awareness campaigns about eye care particularly the importance of annual eye examinations and the consequences of delayed eye examination be made in the form of billboards, mainstream media and social media.

## Figures and Tables

**FIGURE 1-A1:**
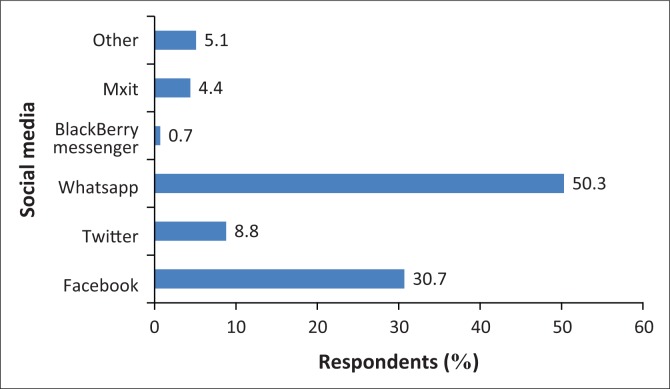
Parents who reported access to social media.

**FIGURE 1-A3:**
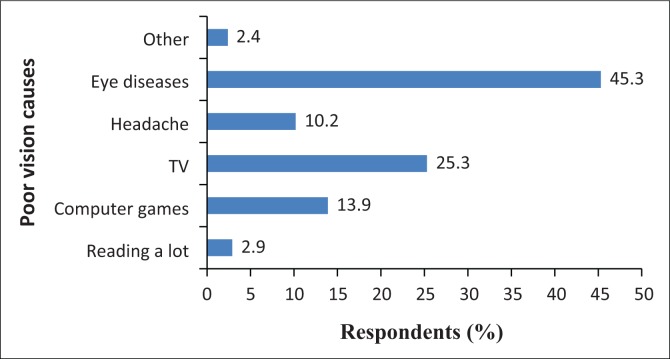
Parents responses regarding the causes of poor vision reported.

**FIGURE 1-A5:**
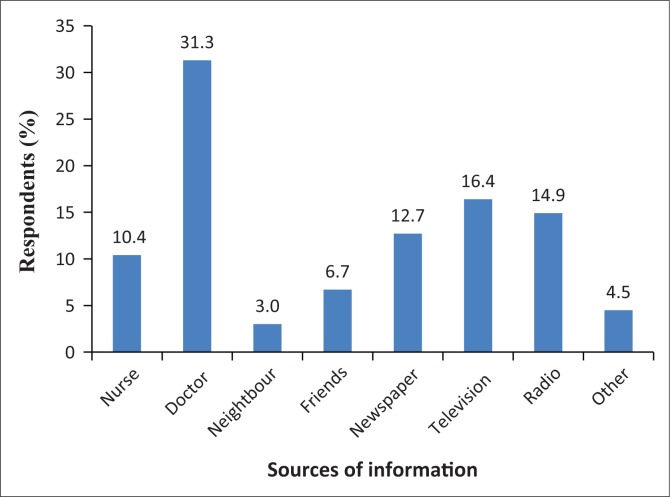
Parents responses regarding sources of information about eye conditions.

## Appendix 2

**TABLE 1-A2:** Parents responses regarding reasons for taking their children to see an eye care practitioner.

Variables	What made you decide to take your child and/or children to see an eye care practitioner?		Variables	If you observed something that prompted you to take your child to an eye care practitioner, what was it?	
	*n*	%		*n*	%
Informed by teacher	7	10.1	Child plays with toys at close range	3	4.5
Advised over radio	2	2.9	Rubbing eyes repeatedly	38	56.7
Advised in newspaper	5	7.3	Tilting the head to one side	-	-
Suspected vision problem	28	40.6	Excessive clumsiness	2	3.0
Child born with vision problem	-	-	Headache	15	22.4
Doctor referred me	18	26.1	Nausea or dizziness	1	1.5
Nurse referred me	5	7.3	Squinting of eyes	8	11.9
Other	4	5.8	-	-	-

## Appendix 4

**TABLE 1-A4:** Responses of parents regarding knowledge of eye conditions affecting children.

Question and/or Item	Yes		No	
	*n*	%	*n*	%
Do you know of any eye diseases that affects children?	76	43.9	97	53.1
If yes, which of the following eye diseases do you know?	-	-	-	-
Congenital cataract	16	11.9	-	-
Trachoma	5	3.7	-	-
Allergic conjunctivitis	37	27.6	-	-
Vitamin A deficiency	26	19.4	-	-
Refractive error (short sighted and far sightedness)	44	32.8	-	-
None of the mentioned diseases	12	4.5	-	-

## Appendix 6

**TABLE 1-A6:** Responses of parents regarding official visits in communities by eye health professionals.

Question and/or Item	Yes		No		Unsure	
	*n*	%	*n*	%	*n*	%
Have eye health officials ever visited your area?	39	22.5	102	59.0	32	-
If yes, when was the last time they visited?	-	-	-	-	-	-
Less than one year ago	19	47.5	-	-	-	-
Greater than 1 year ago	7	17.5	-	-	-	-
2–4 years ago	10	25.0	-	-	-	-
More than 5 years ago	4	10.0	-	-	-	-
**Which of the following eye practitioners were present during the visit?**						
Optometrist	19	43.2	-	-	-	-
Ophthalmic nurse	3	6.8	-	-	-	-
Ophthalmologist	3	6.8	-	-	-	-
Allied health workers	10	22.7	-	-	-	-
Never heard of any	9	20.5	-	-	-	-
